# Role of Primary Caregivers Regarding Unintentional Injury Prevention Among Preschool Children: A Cross-Sectional Survey in Low- and Middle-Income Country

**DOI:** 10.7759/cureus.28599

**Published:** 2022-08-30

**Authors:** Abdus Salam, Danish A Aziz, Farrukh Ansar, Aqib Sajjad, Muhammad Asjid

**Affiliations:** 1 General Surgery, Khyber Teaching Hospital, Peshawar, PAK; 2 Pediatrics and Child Health, Aga Khan University Hospital, Karachi, PAK; 3 Internal Medicine, King Abdullah Teaching Hospital, Mansehra, PAK

**Keywords:** paediatrics, public health, counselling, injury prevention, children, unintentional injuries

## Abstract

Importance

Unintentional childhood injuries significantly strain healthcare resources, and their preventable measures can significantly reduce morbidity and mortality.

Objectives

To investigate the role of primary caregivers in preventing unintentional injuries and to identify the groups that require special health intervention programs to reduce the burden of this public health concern.

Methodology

A cross-sectional survey was conducted at three hospitals in Karachi, Pakistan. Parents of preschool children who visited pediatric clinics were invited to participate in the study by completing a self-administered questionnaire comprising questions about knowledge, attitudes, and practices towards preventing unintentional injuries among children.

Results

With an 80% response rate, the overall mean knowledge, attitude, and practices (KAP) score was 27.40 ± 3.48. Only 14.3% of the participants had a high KAP score, while 83.6% and 2.1% of the respondents had moderate and low KAP scores, respectively. People of lower socioeconomic status, unemployed, less educated, and families with more than one preschool child were less knowledgeable and non-adherent to unintentional preventive injury. It was found that 21% of the children had suffered from an unintentional severe injury in the past, and the internet was the most frequent source of gaining knowledge among parents.

Conclusion

Parental knowledge, attitude, practices, and adherence to child safety measures are sub-optimal in our cohort of studied participants. Raising awareness and providing the counseling are essential in reducing the burden of unintentional injuries.

## Introduction

Domestic unintentional injuries in children are one of the leading causes of morbidity and mortality, accounting for an estimated 875,000 deaths yearly [1]. In addition to being the leading cause of death in preschool children, unintentional injuries lead to several lifetime disabilities [2]. Despite being under full-time care and supervision, many unintentional injuries among preschool children occur in or around the home, as their environment contains a lot of potential risk factors for causing injuries [3]. Some significant risks identified are the lack of doors and safety bars on windows and stairs, open water reservoirs, easy access to burners, openly displayed knives, medicines, poisonous substances, and pesticides [4]. Other risk factors include low socioeconomic status, unsafe homes, lack of awareness and knowledge among mothers, and the careless attitude of the parents [5]. Also, due to limited developmental maturity, physical coordination, and cognitive abilities, children under five years of age are more vulnerable to poor judgment regarding their hazards [6].

Historical data shows that in the United States, the mortality rate among preschool children due to unintentional injuries is higher than other childhood illnesses combined [7]. Even though infectious diseases are the primary cause of most of the deaths among preschool children in developing countries, deaths due to unintentional injuries are increasing at an alarming rate [8]. Literature shows that the mortality rate of unintentional injuries in children is 65 and 35 per 100,000 in low- and middle-income countries (LMICs) and high-income countries (HICs), respectively [9]. Likewise, when the rate of disability-adjusted life years (DALYs) is compared, 2398 per 100,000 population DALYs are lost due to unintentional injuries in LMICs compared to 774 DALYs lost per 100,000 population in HICs [10].

In Pakistan, a lower middle-income country in the South Asian region, 35% of the total 220 million population are children between 0-14 years [3]. Literature reports that injury is the fifth leading cause of loss of healthy life and the second major cause of disability among the Pakistani population [11,12]. According to the National Health Survey of Pakistan, the estimated injury rate is 35.2 per 1000 children under 15 years of age [13]. Numerous regional studies have reported that most unintentional injuries among children occur at home [14,15].

To decrease unintended domestic injuries and enhance safety behaviours, the key strategy is to educate the primary caregivers on home safety [16]. Caregivers, such as parents, supervise the activities of less than five years old children and are accountable for their environment. They can take actions to limit the incidence of injuries and falls among children only when they have the necessary knowledge required to make decisions for child safety. Thus, it is crucial to calculate the current knowledge and practices about unintentional domestic injuries among primary caregivers of preschool children. This will allow experts to identify the gaps in awareness, attitudes, and the presence of unhealthy practices for future interventions. Since no prior research on this issue has been undertaken in Pakistan, this study was aimed at determining the level of knowledge, attitude, and self-reported practices (KAP) among primary caregivers of preschool children about unintentional domestic injuries and finding any potential association between them.

## Materials and methods

Study design, setting, and participants

A cross-sectional descriptive study was carried out at pediatric clinics of three hospitals in Pakistan, including Aga Khan University Hospital, Karachi; Aga Khan Hospital, Karimabad, Karachi; and Aga Khan Hospital, Garden, Karachi, Pakistan. The study was carried out for one year (June 2020 to July 2021). The study population comprised primary caregivers of preschool children who presented to pediatric clinics for their children in the hospitals mentioned above in Karachi, Pakistan. The primary caregivers responsible for the children's financial support and medical decisions were included in the study. They must have had at least one child under the age of five fill in the questionnaire and be at least 18 years old. Individuals who were not the primary caregivers or primary caregivers of children beyond five and those who refused to participate in the study were excluded. In cases where relatives accompany the child, or family members who do not financially support them or make medical decisions for them were also deemed ineligible for the study. The sample size was calculated using Openepi®, assuming a prevalence of 50% of unintentional childhood injuries in children under five since there are no previous estimates available in Pakistan. Keeping the confidence interval at 95% and a precision of 5%, the estimated minimum sample size was 384.

Instrument and data collection

A comprehensive literature search was carried out, and a 50-item standardized questionnaire was developed [17,18]. The questionnaire was validated and re-adjusted after a pilot study was conducted on 25 participants-the questionnaire comprised four main sections. The first section included questions about demographic data, while the second, third, and fourth sections were comprised of questions regarding knowledge, attitude, and practices, respectively. There were 11 questions about demographics, 14 questions about knowledge, six questions about attitude, and 19 questions about practices. The maximum attainable score was 39, as each item from knowledge, attitude, and practice was scored one point for a correct answer. Respondents were classified into three categories based on their scores: those who obtained a KAP score above 70% were considered well-aware (high level). In contrast, scores between 50% and 70% were labeled moderately aware (moderate level), and a score below 50% was considered a lack of awareness (low level) [18].

Research assistants who had received prior training approached the primary caregivers of children in the waiting areas of the pediatrics clinics of the hospitals included in the study and invited them to participate after explaining the nature and purpose of the study. Each participant signed a written consent form and was given a printed copy of the bilingual (English and Urdu) questionnaire. In case of refusal to sign the consent form, the respondent was excluded from the study without making a second attempt to obtain the consent. This was done so that they did not feel pressurized to undertake a study while seeking medical care for their dependent.

Ethical consideration

The Ethics Review Committee (ERC) at Aga Khan University Hospital in Karachi, Pakistan, granted ethical approval for this study (Ref No: 4474). All participants in the research were guaranteed complete confidentiality and anonymity. All participants were given the right to withdraw from the study at any point, and participants received no monetary compensation for their contribution.

Data analysis

Data coding, entry, and analysis were done using SPSS version 23.0 (SPSS Inc., Chicago, IL). Research assistants performed data entry and were cross-checked by other members for potential errors. Descriptive statistics were presented as means, standard deviations, and percentages where appropriate. To determine the normality of the data, the Shapiro-Wilk and Kolmogorov-Smirnov tests were used. Pearson's chi-squared test, student's t-test, and multinomial regression model were used for statistical analysis. A p-value of < 0.05 and a 95% confidence interval was considered significant for statistical significance.

## Results

A total of 480 primary caregivers were approached to participate in the study, of which 384 participants agreed to participate, yielding a response rate of 80%. The participant's mean age was 32.63 ± 5.83 years (range: 22 to 55). The sociodemographic characteristics of the participants are shown in Table 1.

**Table 1:** The sociodemographic characteristics of the primary caregivers who participated in the survey (N=384)

Characteristics	Frequency (n=384)	Percentage
Age		
Less than 30 years	162	42.2%
More than 30 years	222	57.8%
Gender distribution		
Female	206	53.6%
Male	178	46.4%
Marital status		
Married	352	91.7%
Separated/Single	32	8.3%
Educational status		
Primary Education	7	1.8%
Matriculation	19	4.9%
A Levels/Equivalent	51	13.3%
Graduation	307	79.9%
Occupational status		
Corporate Job	161	41.9%
Own Business	45	11.7%
Health Care provider	30	7.8%
Unemployed	24	6.2%
Housewife/husband	124	32.3%
Family Income (in Pakistani Rupees)		
Less than 25000	84	21.9%
26000 to 50000	95	24.7%
51000 to 100000	90	23.4%
100000 to 200000	61	15.9%
Above 2000000	54	14.1%
Number of family members		
Less than four	117	30.5%
More than four	267	69.5%
Number of children less than the age of five		
Less than two	211	54.9%
Two or more	173	45.1%
Relation of participants to child		
Mother	206	53.6%
Father	178	46.4%

Table 2 shows the cross-tabulation of KAP score categories across different variables. The mean-KAP score was 27.40 ± 3.48 (range: 15 to 35). Out of the study population, 55 (14.3%) participants scored 30 or higher. They were considered individuals with a high KAP score; 321 (83.6%) participants scored between 20 and 30 and were labeled as having a moderate KAP score. In comparison, 8 (2.1%) individuals scored less than 20 and were classified as having a low KAP score.

**Table 2:** Cross-tabulation of the levels of KAP score with demographic characteristics *KAP = Knowledge, attitude, and practices **χ = Pearson’s Chi-square value ***Significant P-value < 0.05

Characteristics	Frequency (n=384)	High KAP* Score	Moderate KAP Score	Low KAP Score	χ**-value	p-value
Age						
Less than 30 years	162 (42.2%)	16	146	0	11.13	0.004***
More than 30 years	222 (57.8%)	39	175	8		
Gender distribution						
Female	206 (53.6%)	30	174	8	7.158	0.031***
Male	178 (46.4%)	25	147	0		
Marital status						
Married	352 (91.7%)	51	293	8	0.873	0.647
Separated/Single	32 (8.3%)	4	28	0		
Educational Status						
Primary Education	7 (1.8%)	0	4	3	38.56	0.001***
Matriculation	19 (4.9%)	7	12	0		
A Levels/Equivalent	51 (13.3%)	3	44	4		
Graduation	307 (79.9%)	42	261	4		
Occupational Status						
Corporate Job	161 (41.9%)	18	135	8	25.00	0.015***
Own Business	45 (11.7%)	9	36	0		
Health Care provider	30 (7.8%)	0	30	0		
Unemployed	24 (6.2%)	3	21	0		
Housewife/husband	124 (32.3%)	25	99	0		
Family Income (PKR)						
Less than 25000	84 (21.9%)	16	68	0	21.84	0.016***
26000 to 50000	95 (24.7%)	4	87	4		
51000 to 100000	90 (23.4%)	12	74	4		
100000 to 200000	61 (15.9%)	13	48	0		
Above 2000000	54 (14.1%)	10	41	0		
Number of Family Members						
Less than four	117 (30.5%)	24	93	0	16.29	0.003***
More than four	267 (69.5%)	31	228	8		
Number of Children less than age of five						
Less than two	211 (54.9%)	20	191	0	29.26	0.001***
Two or more	173 (45.1%)	35	130	8		

Knowledge score of primary caregivers about unintentional injuries

Among primary caregivers of preschool children, the mean score was 11.28 ± 1.69 out of 14 (range: 5 to 14). The top three questions, to which most participants correctly responded, were: "Keeping medicines out of children's reach can prevent accidents" (95.8%), "Toys with small parts can harm children" (95.5%), and "Water heaters can cause injuries to children" (94.1%). While the top three questions on which the least number of participants were able to choose the correct answer were: "Children can be injured by falling out of bed" (32.5%), "Leaving a child unattended in one-foot-deep water can be hazardous," (39.8%) and "Covering children mouth and head with a thick blanket during sleep can cause suffocation" (61.7%). As shown in Table 3, single parents had somewhat more knowledge than married parents (p-value = 0.022), those with a bachelor's degree (p-value = 0.001), and those with a high monthly income (p-value = 0.001). Respondents from households with fewer than four members and parents with only one kid under the age of five performed better on the knowledge scale, albeit no statistical significance was found.

**Table 3:** Distribution of KAP Scores of participating primary caregivers according to sociodemographic characteristics *KAP=Knowledge, attitude, and practices **SD=Standard deviation ***Significant P-value < 0.05

Characteristics	KAP* Score (SD*)	Knowledge Score (SD)	Practices Score (SD)	Attitude Score (SD)
Age				
Less than 30 years	27.6 (2.5)	11.3 (1.3)	12.4 (1.8)	3.95 (1.1)
More than 30 years	27.1 (3.9)	11.2 (1.9)	12.1 (2.2)	3.9 (1.2)
p-value	0.163	0.714	0.933	0.068
Gender distribution				
Female	27.6 (3.1)	11.3 (1.4)	12.2 (2.2)	4.1 (1.2)
Male	27.2 (3.8)	11.2 (1.8)	12.1 (2.1)	3.8 (1.2)
p-value	0.263	0.847	0.064	0.577
Marital status				
Married	27.4 (3.4)	11.2 (1.7)	12.2 (2.2)	3.9 (1.2)
Separated/Single	26.6 (3.5)	11.9 (1.3)	10.8 (2.4)	3.8 (1.4)
p-value	0.207	0.022	0.613	0.001
Educational status				
Primary education	25.0	10.0	11.0	4.0
Matriculation	28.6 (3.1)	11.1 (1.7)	12.8 (1.3)	3.8 (0.3)
A Levels/Equivalent	25.5 (4.4)	10.8 (2.7)	11.5 (2.1)	3.1 (1.5)
Graduation	27.5 (3.2)	11.3 (1.4)	12.2 (2.3)	4.1 (1.1)
p-value (F)	0.001 (5.4)	0.001 (5.5)	0.001 (6.5)	0.127 (1.8)
Occupational status				
Corporate job	26.5 (4.2)	10.9 (1.9)	11.9 (2.5)	3.6 (1.3)
Own business	28.9 (2.2)	11.9 (1.3)	12.7 (2.1)	4.2 (0.8)
Health care provider	27.8 (1.1)	11.1 (1.4)	12.3 (1.4)	4.4 (0.6)
Unemployed	22.1 (0.8)	11.0 (0.8)	8.20 (1.5)	2.8 (1.5)
Housewife/husband	28.1 (2.7)	11.3 (1.5)	12.5 (1.9)	4.1 (1.2)
p-value (F)	0.001 (9.2)	0.001 (4.1)	0.001 (5.1)	0.001 (7.2)
Family income (in Pakistani Rupees)				
Less than 25000	25.2 (3.6)	11.3 (1.5)	9.9 (2.5)	3.1 (1.1)
26000 to 50000	26.7 (3.9)	11.1 (2.1)	12.1 (2.4)	3.5 (1.3)
51000 to 100000	27.2 (3.7)	10.7 (1.3)	12.2 (2.1)	4.1 (1.3)
100000 to 200000	28.1 (2.9)	11.1 (1.6)	12.3 (2.2)	4.5 (0.9)
Above 2000000	28.7 (2.5)	11.5 (1.4)	12.8 (2.1)	4.3 (0.9)
p-value (F)	0.001 (5.4)	0.001 (5.9)	0.001 (10.5)	0.001 (6.1)
Number of family members				
Less than four	28.1 (2.9)	11.4 (1.3)	12.6 (2.1)	4.1 (1.1)
More than four	26.6 (3.7)	11.1 (1.8)	11.7 (2.2)	3.7 (1.3)
p-value	0.001	0.317	0.001	0.001
Number of children less than the age of five				
Less than two	27.6 (2.7)	11.1 (1.7)	12.2 (1.8)	4.1 (1.1)
Two or more	27.1 (3.7)	11.3 (1.8)	12.0 (2.2)	3.6 (1.3)
p-value	0.001***	0.317	0.001***	0.001***

Attitude score of primary caregivers about unintentional injuries

The attitude score was 3.95 ± 1.24 out of 6 (range: 1 to 6). Primary caregivers were most likely to agree with the statement "Unintentional injuries among children may be averted" (64.1%), while the least likely opinion was "Having a first aid kit at home is required to handle accidents" (15.4%). Being married (p-value = 0.001), working in the healthcare industry (p-value = 0.001), earning a high monthly salary (p-value = 0.001), having fewer than four family members (p-value = 0.001), or having just one kid under the age of five (p-value = 0.001) were all linked with a higher attitude score, as shown in Table 3.

Practices score of primary caregivers about unintentional injuries

The mean practice score was 12.16 ± 2.26 out of the maximum attainable score of 19 (range: 5 to 18). The top three least complying practices were "Use of safety barriers in the kitchen/near burners" (33.3%), "Not covering the baby's head/mouth during sleep" (37.5%), and "Do not let the children play with small items/toys" (46.3%). While the most compliant practices were "Not allowing the child to play alone in the bathtub (83.6%), "Feeding the child under continual supervision (80.2%), and "Keeping medicines locked away on the top shelf" (73.4%). Caregivers who owned a business (p-value = 0.001), had a high income (p-value = 0.001), had fewer than four family members (p-value = 0.001), or had just one kid under the age of five (p-value = 0.001) performed significantly better on practice scale, as shown in Table 3.

Predictors of high KAP score

As shown in Table 4, a multinomial regression model did not reveal any significant difference in high KAP scores between the male and female gender. Married parents performed better than single or separated parents (OR = 1.18, p-value = 0.759). Those with a graduation degree or equivalent had a significantly higher KAP score than those who had only primary (OR = 0.211, p-value = 0.027) or secondary education (OR = 0.272, p-value = 0.010). Being a healthcare service provider was also a positive predictive factor towards a high KAP score (OR = 3.81, p-value = 0.001) while being unemployed resulted in a low KAP score (OR = 0.76, p-value = 0.386). A low family income appeared to impede achieving a high KAP score (OR = 0.18, p-value = 0.006). Primary caregivers in families with one child under the age of five outperformed those in families with more than one child under the age of five on the KAP scale (OR = 2.42, p-value = 0.003).

**Table 4:** Association between important variables and KAP scores (high vs. low) using multinomial regression model *B=coefficient **Wald=Wald statistics ***Significant P-value < 0.05 ****CI=Confidence interval

Variables	B*	Wald**	p-value	Odds ratio (OR)	95% CI**** for OR		
					Upper	Lower	
Gender distribution							
Male	0.031	0.011	0.915	0.969	0.546	1.72	
Female	Reference						
Marital status							
Married	0.171	0.094	0.759	1.18	0.399	3.52	
Separated/Single	Reference						
Educational status							
Primary education	1.55	3.95	0.027***	0.211	0.046	0.978	
Matriculation	1.31	6.69	0.010***	0.272	0.101	0.729	
A Levels/Equivalent	0.931	2.26	0.132	2.53	0.755	8.51	
Graduation	Reference						
Occupational status							
Corporate job	0.696	4.32	0.038***	2.01	1.03	3.87	
Own business	0.010	0.435	0.982	1.01	0.431	2.36	
Health care provider	20.6	37.8	.001***	3.81	2.12	6.38	
Unemployed	0.570	0.753	0.386	0.76	0.488	6.41	
Housewife/husband	Reference						
Family income (in Pakistani Rupees)							
Less than 25000	1.71	7.61	0.006***	0.180	0.053	0.608	
26000 to 50000	0.461	0.963	0.326	0.631	0.251	1.58	
51000 to 100000	0.079	0.031	0.860	0.924	0.384	2.22	
100000 to 200000	0.105	0.049	0.824	1.10	0.441	2.79	
Above 2000000	Reference						
Number of family members							
Less than four	-0.675	5.12	0.024***	0.509	0.284		0.913
More than four	Reference						
Number of children less than the age of five							
Less than two	0.885	8.59	0.003***	2.42	1.34		4.24
Two or more	Reference						

Source of knowledge

Participants responded that the primary source of knowledge regarding the prevention of unintentional injuries among children was the internet (27.9%), followed by advice from family members or relatives (26%), healthcare service providers (21.9%), electronic media (14.8%), and print media (including books) (9.4%). The highest KAP score (28.22 ± 1.1) was observed among those who relied on print media to obtain necessary information regarding preventing unintentional injuries, followed by those who consulted healthcare service providers for advice (27.32 ± 3.4). In contrast, the lowest score was observed among those who relied on electronic media (25.98 ± 5.1).

## Discussion

Preschool children's injuries can have long-term consequences, increasing morbidity and death. Unintentional injuries among children are a significant concern, with an estimated 6.16 million unintentional injuries occurring each year in Pakistan among preschool children, and the number is increasing [19]. As a developing country, Pakistan struggles to maintain a decent healthcare system in terms of quality and availability, with only 3% of its GDP dedicated to total healthcare expenditures, making it difficult for the health system to provide proper short-and long-term treatment and rehabilitation to patients suffering from unintentional injuries. By minimizing the impact of unintentional injuries, we can help redirect health resources to deal with unpreventable illnesses. It is critical to assess primary caregivers' knowledge, attitudes, and practices regarding unintentional injuries to better understand the level of awareness and to identify specific groups lacking awareness based on socioeconomic characteristics to plan and implement effective interventions.

The current study discovered that various characteristics affect high KAP compliance, such as parents who live together, educational status, family income, occupation of parents, small family size, and having fewer children under the age of five. Another Turkish study indicated that parental educational and occupational status, the number of dependents in a house, and the child's age were all associated with adherence to unintentional injury prevention [20].

As we found that the educational status of the primary caregivers was a significant factor associated with the parents' adherence to unintentional injury prevention, parents with an A-level or comparable education were substantially more likely to comply with unintentional injury prevention than those with only elementary or matriculation education. A qualitative study from Europe also emphasized that injury recognition capacity is directly proportional to the mother's education level [21]. Another European study concluded that the risk of unintentional injury among the children of parents who had only primary education was 1.5 times higher than in those children whose parents had a graduate degree. Similar results were found in our study, stating that compliance with unintentional injury prevention among parents with an elementary education was 80% lower than those with a college degree [22]. The occupational status of primary caregivers was also a prime factor in the risk of averting unintentional injuries in preschool children. Our findings suggest that, compared to homemakers or husbands, parents who work in the healthcare sector were 3.8 times more vigilant in preventing unintentional injuries among their children. A Swedish study reported that having a health care expert in a household is associated with better preventative health care [23]. Our findings show that jobless parents scored lower on the KAP scale than all other occupational categories. Unemployment can cause financial restraints and negative consequences for a parent's psychological well-being, making them less likely to make vital decisions for their child's health. A Slovakian study reported that a father's long-term unemployment might negatively influence his child's health [24].

Our study also found a significant relationship between family income and KAP score. Primary caregivers earning the minimum wage were 80 percent less compliant with unintentional injury prevention measures than those earning three to four times the minimum wage. Another study supports our findings by indicating that, even after adjusting for other factors, lower general health and more excellent hospital admission rates were associated with lower family income [25]. Literature shows that children growing up in low-income families have poorer health than those from higher-income families and perform relatively poorly on psychosocial, intellectual, and developmental evaluations [26]. A high-quality meta-analysis revealed that a decent family income had an evident positive causal influence on variables crucial for children's well-being and development, such as safe parenting, a healthy environment, and the mental health of primary caregivers [27]. Low parental adherence to safety measures in low-income families may be attributed to financial constraints that prevent them from prioritizing safety items and actions that need financial allocation.

Medicines, prescription and over-the-counter (OTC), are the most common types of products implicated in unintentional poisoning, with children under five being the most vulnerable [28]. A study of secondary data from the American Association of Poison Control Centers (2000 to 2009) found that an increase in the use of anti-diabetic medicines, anti-hypercholesterolemia drugs, antihypertensive, and pain relievers was associated with an increase in cases of unintentional child poisoning, leading to higher hospital admissions [29]. The National Electronic Injury Surveillance System -- Cooperative Adverse Drug Event Surveillance Project (NEISS-CADES) data were used to assess trends in emergency department visits for unsupervised medication exposures in children under the age of six years, and it was discovered that easy access to medicines and a lack of supervision were major factors [30]. In our study, only 73% of the parents kept medicines away from their children on the top shelf or locked in a drawer. According to a Turkish study, 80 percent of the sample population kept medications and poisons out of children's reach [20]. However, a Brazilian study indicated that almost 21 percent of the study sample did not keep medicines and poisonous substances in a secure area away from children [28]. To prevent this detrimental behavior and minimize child poisoning mortality, enhanced parental awareness about the risk of poisoning is critical, as is regulating child-resistant packaging of medications and toxic chemicals, which can significantly reduce the risk of inadvertent poisoning. According to our findings, only 22% of primary caregivers sought advice from physicians or other professional health care workers about preventing unintentional injuries in their children. This demonstrates that healthcare facilities are not a significant source of raising parental awareness. Many children visit general practitioners and pediatricians for vaccinations, minor ailments, and other health-related issues, providing an excellent opportunity for healthcare professionals to incorporate proper coaching, child safety programs, and interventions to promote child safety practices at the primary level.

Limitations

The cross-sectional study design and small sample size do not allow us to make assumptions about causal relationships between parameters; therefore, generalizability may be limited. Secondly, the sample was drawn from only three hospitals in a single large urban city, providing room for selection bias. Lastly, data collection through a face-to-face questionnaire might lead to social desirability and recall bias.

Future directions

A population-based cross-sectional study with household inspection for essential preventable injury items could give a better insight into the extent of adherence by primary caregivers. Furthermore, practices vary from region to region; a country-wide randomized sampling or cluster sampling of households would give an actual estimate.

## Conclusions

In Pakistan, primary caregivers of preschool children have suboptimal knowledge, attitudes, and practices for preventing unintentional injuries. People of lower socioeconomic status, the unemployed, the less educated, and prominent families with more than one preschool child are less likely to comply with unintentional injury preventive measures. These groups should be specifically targeted for intervention programs. Primary health care professionals can play a beneficial role in preventing childhood injuries by introducing child safety programs and interventions to promote child safety practices among primary caregivers who present to hospitals and clinics.
